# 2-{[(Pyridin-2-yl)amino]­meth­yl}phenol

**DOI:** 10.1107/S1600536812031340

**Published:** 2012-07-18

**Authors:** Shan Gao, Seik Weng Ng

**Affiliations:** a Key Laboratory of Functional Inorganic Materials Chemistry, Ministry of Education, Heilongjiang University, Harbin 150080, People’s Republic of China; b Department of Chemistry, University of Malaya, 50603 Kuala Lumpur, Malaysia; c Chemistry Department, Faculty of Science, King Abdulaziz University, PO Box 80203, Jeddah, Saudi Arabia

## Abstract

The planes of the aromatic rings of the title compound, C_12_H_12_N_2_O, are twisted by 50.33 (15)°. The phenol O atom is a hydrogen-bond donor to the pyridine N atom, resulting in the formation of an eight-membered ring in the mol­ecule. The amino N atom is a hydrogen-bond donor to the phenol O atom of an adjacent mol­ecule; this hydrogen bond leads to the formation of a helical chain that runs along the *a* axis.

## Related literature

For the related compound 2-{[(pyrazin-2-yl)amino]­meth­yl}­phenol, see: Gao & Ng (2012 ▶). For 2-[(pyridin-3-yl­amino)­meth­yl]phenol, see: Xu *et al.* (2011 ▶). For the metal adducts of 2-[(pyridin-2-yl­amino)­meth­yl]phenol, see: Yalçın *et al.* (2007 ▶).
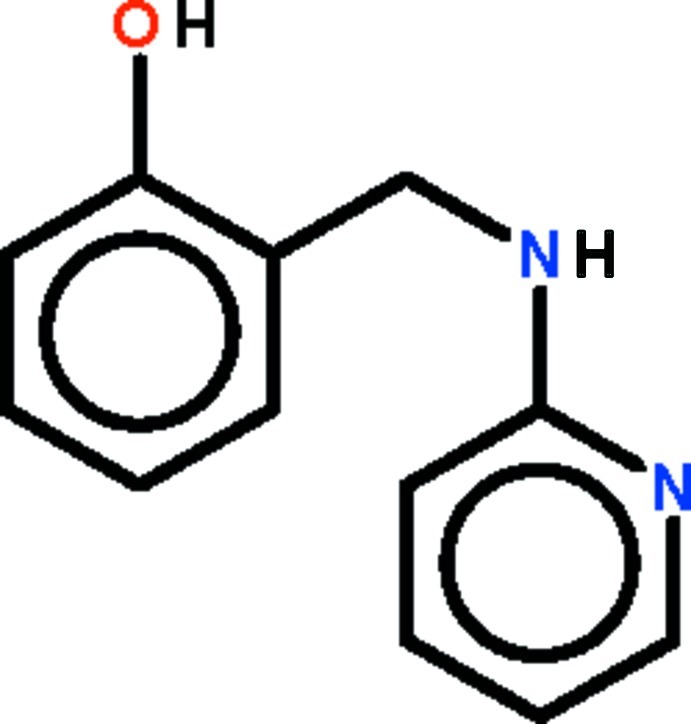


## Experimental

### 

#### Crystal data

C_12_H_12_N_2_O
*M* *_r_* = 200.24
Orthorhombic, 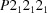

*a* = 6.3331 (4) Å
*b* = 10.6761 (9) Å
*c* = 15.3714 (10) Å
*V* = 1039.30 (13) Å^3^
*Z* = 4
Mo *K*α radiation
μ = 0.08 mm^−1^
*T* = 295 K
0.25 × 0.19 × 0.15 mm

#### Data collection

Rigaku R-AXIS RAPID IP diffractometer
Absorption correction: multi-scan (*ABSCOR*; Higashi, 1995 ▶) *T*_min_ = 0.979, *T*_max_ = 0.988
10221 measured reflections
1391 independent reflections
887 reflections with *I* > 2σ(*I*)
*R*_int_ = 0.047

#### Refinement

*R*[*F* ^2^ > 2σ(*F* ^2^)] = 0.043
*wR*(*F* ^2^) = 0.131
*S* = 1.04
1391 reflections
144 parameters
2 restraints
H atoms treated by a mixture of independent and constrained refinement
Δρ_max_ = 0.13 e Å^−3^
Δρ_min_ = −0.18 e Å^−3^

### 

Data collection: *RAPID-AUTO* (Rigaku, 1998 ▶); cell refinement: *RAPID-AUTO*; data reduction: *CrystalClear* (Rigaku/MSC, 2002 ▶); program(s) used to solve structure: *SHELXS97* (Sheldrick, 2008 ▶); program(s) used to refine structure: *SHELXL97* (Sheldrick, 2008 ▶); molecular graphics: *X-SEED* (Barbour, 2001 ▶); software used to prepare material for publication: *publCIF* (Westrip, 2010 ▶).

## Supplementary Material

Crystal structure: contains datablock(s) global, I. DOI: 10.1107/S1600536812031340/xu5581sup1.cif

Structure factors: contains datablock(s) I. DOI: 10.1107/S1600536812031340/xu5581Isup2.hkl

Supplementary material file. DOI: 10.1107/S1600536812031340/xu5581Isup3.cml

Additional supplementary materials:  crystallographic information; 3D view; checkCIF report

## Figures and Tables

**Table 1:** Hydrogen-bond geometry (Å, °)

*D*—H⋯*A*	*D*—H	H⋯*A*	*D*⋯*A*	*D*—H⋯*A*
O1—H1⋯N1	0.85 (1)	1.83 (2)	2.658 (3)	166 (5)
N2—H2⋯O1^i^	0.88 (1)	2.06 (1)	2.928 (3)	172 (3)

Symmetry code: (i) 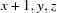.

**Fig. 1.**
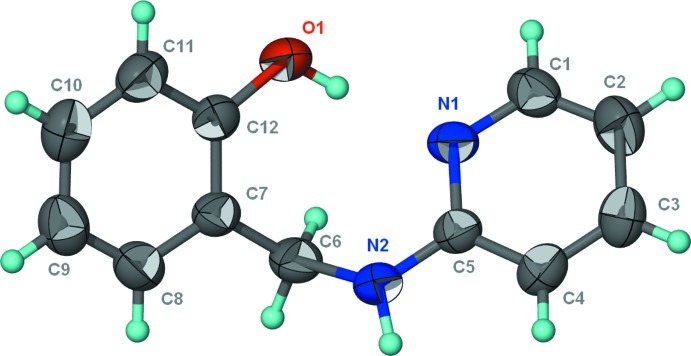
Thermal ellipsoid plot (Barbour, 2001) of C12H12N2O at the 50% probability level; hydrogen atoms are drawn as spheres of arbitrary radius.

**Fig. 2.**
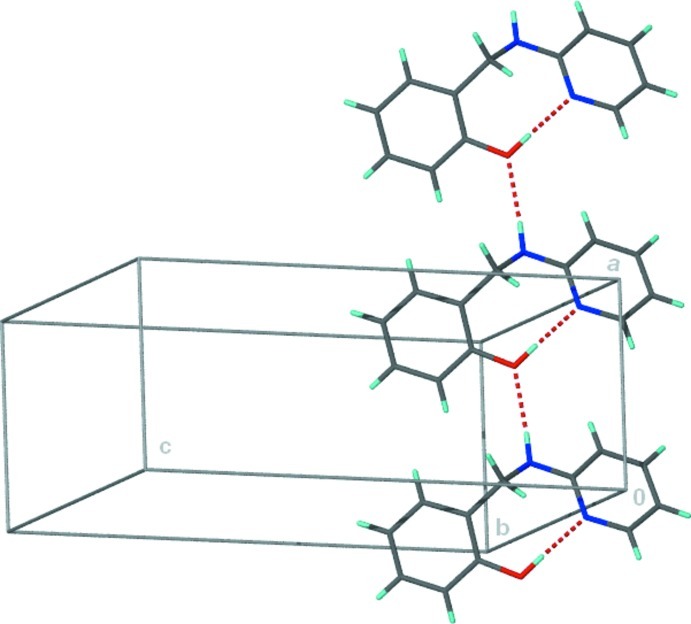
Hydrogen-bonded chain motif.

## supplementary crystallographic information

### Comment

Salicylaldehyde condenses with aromatic amines to yield Schiff bases, which serve as chelating ligands to a plethora of metal systems. These Schiff bases can be readily reduce to the corresponding secondary amines, which can also function as chelating ligands. Curiously, there are only few 2-(arylamino)methylphenols compared with the plethora of Schiff bases in the chemical literature. Among the aminopyridine derivatives, only the crystal structure of 2-((pyridin-3-ylamino)methyl)phenol has been reported (Xu *et al.*, 2011). The 2-((pyridin-2-ylamino)methyl)phenol analog (Scheme I) has been described as its metal adducts only (Yalçın *et al.*, 2007).

The two aromatic rings of the reduced Schiff-base, C_12_H_12_N_2_O, are twisted along the –CH_2_–NH– single-bond by 50.3 (1) °. The hydroxy O atom is hydrogen-bond donor to the pyridyl N atom and an eight-membered ring is formed (Fig. 1). The slightly flattened secondary amino N atom is hydrogen-bond donor to the O atom of an adjacent molecule; this hydrogen bond leads to the formation of a helical chain that runs along the *a*-axis of the orthorhombic unit cell (Fig. 2, Table 1).

### Experimental

A solution of 2-aminopyridine (1 mmol) and salicylaldehyde (1 mmol) in toluene (50 ml) was heated for 10 h. The solvent was removed under vacuum, and the residue was reduced in absolute methanol by sodium borohydride. Light yellow crystals were obtained by recrystallization from methanol in 80% yield.

### Refinement

Carbon-bound H-atoms were placed in calculated positions (C–H 0.93 Å) and were included in the refinement in the riding model approximation, with *U*(H) set to 1.2*U*(C). The amino and hydroxy H-atoms were located in a difference Fourier map, and were refined with distance restraints N–H 0.88±0.01 Å and O–H 0.84±0.01 Å; their temperature factors were refined.

In the absence of heavy scatters, 980 Friedel pairs were merged.

### Crystal data

**Table:**

C_12_H_12_N_2_O	*F*(000) = 424
*M**_r_* = 200.24	*D*_x_ = 1.280 Mg m^−^^3^
Orthorhombic, *P*2_1_2_1_2_1_	Mo *K*α radiation, λ = 0.71073 Å
Hall symbol: P 2ac 2ab	Cell parameters from 6231 reflections
*a* = 6.3331 (4) Å	θ = 3.3–27.4°
*b* = 10.6761 (9) Å	µ = 0.08 mm^−^^1^
*c* = 15.3714 (10) Å	*T* = 295 K
*V* = 1039.30 (13) Å^3^	Prism, faint yellow
*Z* = 4	0.25 × 0.19 × 0.15 mm

### Data collection

**Table:**

Rigaku R-AXIS RAPID IP diffractometer	1391 independent reflections
Radiation source: fine-focus sealed tube	887 reflections with *I* > 2σ(*I*)
Graphite monochromator	*R*_int_ = 0.047
ω scan	θ_max_ = 27.4°, θ_min_ = 3.3°
Absorption correction: multi-scan (*ABSCOR*; Higashi, 1995)	*h* = −8→8
*T*_min_ = 0.979, *T*_max_ = 0.988	*k* = −13→13
10221 measured reflections	*l* = −19→19

### Refinement

**Table:**

Refinement on *F*^2^	Primary atom site location: structure-invariant direct methods
Least-squares matrix: full	Secondary atom site location: difference Fourier map
*R*[*F*^2^ > 2σ(*F*^2^)] = 0.043	Hydrogen site location: inferred from neighbouring sites
*wR*(*F*^2^) = 0.131	H atoms treated by a mixture of independent and constrained refinement
*S* = 1.04	*w* = 1/[σ^2^(*F*_o_^2^) + (0.0773*P*)^2^] where *P* = (*F*_o_^2^ + 2*F*_c_^2^)/3
1391 reflections	(Δ/σ)_max_ = 0.001
144 parameters	Δρ_max_ = 0.13 e Å^−^^3^
2 restraints	Δρ_min_ = −0.18 e Å^−^^3^

### Fractional atomic coordinates and isotropic or equivalent isotropic displacement parameters (Å^2^)

**Table:**

	*x*	*y*	*z*	*U*_iso_*/*U*_eq_	
O1	0.6052 (3)	0.1233 (2)	0.18887 (14)	0.0752 (7)	
N1	0.8839 (3)	0.0670 (2)	0.06566 (14)	0.0570 (6)	
N2	1.1470 (4)	0.1185 (3)	0.16368 (15)	0.0672 (8)	
C1	0.8251 (6)	0.0489 (3)	−0.01804 (18)	0.0672 (8)	
H1A	0.6882	0.0212	−0.0286	0.081*	
C2	0.9537 (6)	0.0687 (3)	−0.0877 (2)	0.0762 (9)	
H2A	0.9069	0.0534	−0.1441	0.091*	
C3	1.1575 (6)	0.1126 (3)	−0.0720 (2)	0.0706 (9)	
H3	1.2483	0.1294	−0.1181	0.085*	
C4	1.2220 (5)	0.1305 (3)	0.01124 (19)	0.0636 (8)	
H4	1.3573	0.1600	0.0227	0.076*	
C5	1.0823 (4)	0.1041 (3)	0.08020 (17)	0.0542 (7)	
C6	1.0371 (5)	0.0617 (3)	0.23756 (18)	0.0652 (8)	
H6A	1.1414	0.0269	0.2769	0.078*	
H6B	0.9513	−0.0071	0.2163	0.078*	
C7	0.8978 (4)	0.1498 (3)	0.28793 (17)	0.0564 (7)	
C8	0.9675 (5)	0.2047 (3)	0.36466 (18)	0.0672 (9)	
H8	1.1032	0.1872	0.3843	0.081*	
C9	0.8415 (6)	0.2848 (3)	0.4129 (2)	0.0761 (9)	
H9	0.8911	0.3193	0.4645	0.091*	
C10	0.6432 (6)	0.3126 (3)	0.3835 (2)	0.0763 (10)	
H10	0.5583	0.3673	0.4150	0.092*	
C11	0.5680 (5)	0.2601 (3)	0.30782 (18)	0.0692 (8)	
H11	0.4334	0.2801	0.2881	0.083*	
C12	0.6935 (4)	0.1769 (3)	0.26072 (17)	0.0567 (7)	
H1	0.694 (5)	0.093 (4)	0.153 (2)	0.119 (17)*	
H2	1.2851 (18)	0.126 (4)	0.167 (2)	0.092 (11)*	

### Atomic displacement parameters (Å^2^)

**Table:**

	*U*^11^	*U*^22^	*U*^33^	*U*^12^	*U*^13^	*U*^23^
O1	0.0449 (10)	0.1083 (19)	0.0723 (13)	−0.0019 (12)	−0.0022 (11)	−0.0175 (13)
N1	0.0441 (11)	0.0614 (15)	0.0655 (13)	0.0003 (11)	−0.0053 (11)	−0.0049 (11)
N2	0.0426 (12)	0.099 (2)	0.0599 (14)	−0.0044 (13)	−0.0005 (11)	−0.0014 (13)
C1	0.0658 (18)	0.068 (2)	0.0680 (18)	−0.0014 (15)	−0.0109 (16)	−0.0112 (15)
C2	0.094 (2)	0.073 (2)	0.0620 (16)	−0.001 (2)	−0.0054 (19)	−0.0145 (16)
C3	0.084 (2)	0.064 (2)	0.0638 (18)	0.0032 (17)	0.0141 (16)	−0.0084 (14)
C4	0.0585 (16)	0.0609 (19)	0.0715 (19)	−0.0017 (15)	0.0113 (15)	−0.0067 (14)
C5	0.0471 (14)	0.0553 (17)	0.0601 (14)	0.0020 (13)	0.0012 (13)	−0.0030 (13)
C6	0.0544 (15)	0.078 (2)	0.0634 (16)	0.0060 (15)	−0.0075 (14)	0.0105 (15)
C7	0.0471 (14)	0.0667 (19)	0.0555 (14)	−0.0064 (13)	0.0017 (13)	0.0094 (13)
C8	0.0610 (17)	0.082 (2)	0.0581 (15)	−0.0153 (17)	−0.0026 (15)	0.0086 (15)
C9	0.090 (2)	0.077 (2)	0.0616 (16)	−0.022 (2)	0.0027 (18)	−0.0032 (17)
C10	0.087 (2)	0.071 (2)	0.0709 (18)	−0.0049 (19)	0.0184 (18)	−0.0051 (16)
C11	0.0581 (16)	0.078 (2)	0.0713 (17)	0.0046 (17)	0.0107 (15)	0.0055 (17)
C12	0.0497 (14)	0.0644 (19)	0.0561 (15)	−0.0075 (13)	0.0031 (13)	−0.0002 (13)

### Geometric parameters (Å, º)

**Table:**

O1—C12	1.364 (3)	C4—H4	0.9300
O1—H1	0.851 (10)	C6—C7	1.504 (4)
N1—C5	1.336 (4)	C6—H6A	0.9700
N1—C1	1.353 (3)	C6—H6B	0.9700
N2—C5	1.356 (3)	C7—C8	1.389 (4)
N2—C6	1.464 (4)	C7—C12	1.390 (4)
N2—H2	0.879 (10)	C8—C9	1.384 (5)
C1—C2	1.362 (5)	C8—H8	0.9300
C1—H1A	0.9300	C9—C10	1.367 (5)
C2—C3	1.395 (5)	C9—H9	0.9300
C2—H2A	0.9300	C10—C11	1.377 (4)
C3—C4	1.357 (4)	C10—H10	0.9300
C3—H3	0.9300	C11—C12	1.394 (4)
C4—C5	1.409 (4)	C11—H11	0.9300
			
C12—O1—H1	114 (3)	C7—C6—H6A	108.6
C5—N1—C1	117.4 (3)	N2—C6—H6B	108.6
C5—N2—C6	122.9 (3)	C7—C6—H6B	108.6
C5—N2—H2	111 (2)	H6A—C6—H6B	107.5
C6—N2—H2	118 (2)	C8—C7—C12	117.6 (3)
N1—C1—C2	124.1 (3)	C8—C7—C6	121.0 (3)
N1—C1—H1A	118.0	C12—C7—C6	121.4 (3)
C2—C1—H1A	118.0	C9—C8—C7	122.1 (3)
C1—C2—C3	118.0 (3)	C9—C8—H8	118.9
C1—C2—H2A	121.0	C7—C8—H8	118.9
C3—C2—H2A	121.0	C10—C9—C8	119.1 (3)
C4—C3—C2	119.3 (3)	C10—C9—H9	120.4
C4—C3—H3	120.4	C8—C9—H9	120.4
C2—C3—H3	120.4	C9—C10—C11	120.5 (3)
C3—C4—C5	119.5 (3)	C9—C10—H10	119.7
C3—C4—H4	120.2	C11—C10—H10	119.7
C5—C4—H4	120.2	C10—C11—C12	120.1 (3)
N1—C5—N2	118.4 (2)	C10—C11—H11	120.0
N1—C5—C4	121.6 (3)	C12—C11—H11	120.0
N2—C5—C4	120.0 (2)	O1—C12—C7	122.5 (3)
N2—C6—C7	114.8 (3)	O1—C12—C11	117.0 (3)
N2—C6—H6A	108.6	C7—C12—C11	120.5 (3)
			
C5—N1—C1—C2	−1.4 (5)	N2—C6—C7—C12	82.9 (4)
N1—C1—C2—C3	−1.3 (5)	C12—C7—C8—C9	−0.6 (4)
C1—C2—C3—C4	1.8 (5)	C6—C7—C8—C9	−179.1 (3)
C2—C3—C4—C5	0.2 (5)	C7—C8—C9—C10	−1.1 (5)
C1—N1—C5—N2	−177.7 (3)	C8—C9—C10—C11	1.1 (5)
C1—N1—C5—C4	3.6 (4)	C9—C10—C11—C12	0.6 (5)
C6—N2—C5—N1	18.7 (5)	C8—C7—C12—O1	−176.6 (3)
C6—N2—C5—C4	−162.5 (3)	C6—C7—C12—O1	1.9 (4)
C3—C4—C5—N1	−3.0 (5)	C8—C7—C12—C11	2.3 (4)
C3—C4—C5—N2	178.2 (3)	C6—C7—C12—C11	−179.2 (3)
C5—N2—C6—C7	−101.6 (3)	C10—C11—C12—O1	176.6 (3)
N2—C6—C7—C8	−98.6 (3)	C10—C11—C12—C7	−2.3 (4)

### Hydrogen-bond geometry (Å, º)

**Table:**

*D*—H···*A*	*D*—H	H···*A*	*D*···*A*	*D*—H···*A*
O1—H1···N1	0.85 (1)	1.83 (2)	2.658 (3)	166 (5)
N2—H2···O1^i^	0.88 (1)	2.06 (1)	2.928 (3)	172 (3)

Symmetry code: (i) *x*+1, *y*, *z*.
